# Machine-learning based risk prediction of in-hospital outcomes following STEMI: the STEMI-ML score

**DOI:** 10.3389/fcvm.2024.1454321

**Published:** 2024-10-10

**Authors:** Hari P. Sritharan, Harrison Nguyen, Jonathan Ciofani, Ravinay Bhindi, Usaid K. Allahwala

**Affiliations:** ^1^ Department of Cardiology, Royal North Shore Hospital, Sydney, NSW, Australia; ^2^ Faculty of Medicine and Health, University of Sydney, Sydney, NSW, Australia

**Keywords:** acute coronary syndrome, myocardial infarction, ST-elevation myocardial infarction, machine-learning, risk score

## Abstract

**Background:**

Traditional prognostic models for ST-segment elevation myocardial infarction (STEMI) have limitations in statistical methods and usability.

**Objective:**

We aimed to develop a machine-learning (ML) based risk score to predict in-hospital mortality, intensive care unit (ICU) admission, and left ventricular ejection fraction less than 40% (LVEF < 40%) in STEMI patients.

**Methods:**

We reviewed 1,863 consecutive STEMI patients undergoing primary percutaneous coronary intervention (pPCI) or rescue PCI. Eight supervised ML methods [LASSO, ridge, elastic net (EN), decision tree, support vector machine, random forest, AdaBoost and gradient boosting] were trained and validated. A feature selection method was used to establish more informative and nonredundant variables, which were then considered in groups of 5/10/15/20/25/30(all). Final models were chosen to optimise area under the curve (AUC) score while ensuring interpretability.

**Results:**

Overall, 128 (6.9%) patients died in hospital, with 292 (15.7%) patients requiring ICU admission and 373 (20.0%) patients with LVEF < 40%. The best-performing model with 5 included variables, EN, achieved an AUC of 0.79 for in-hospital mortality, 0.78 for ICU admission, and 0.74 for LVEF < 40%. The included variables were age, pre-hospital cardiac arrest, robust collateral recruitment (Rentrop grade 2 or 3), family history of coronary disease, initial systolic blood pressure, initial heart rate, hypercholesterolemia, culprit vessel, smoking status and TIMI flow pre-PCI. We developed a user-friendly web application for real-world use, yielding risk scores as a percentage.

**Conclusions:**

The STEMI-ML score effectively predicts in-hospital outcomes in STEMI patients and may assist with risk stratification and individualising patient management.

## 1 Introduction

The advent of timely reperfusion strategies for patients with ST elevation myocardial infarction (STEMI) has yielded improvement in prognosis, including mortality (1). Whilst the overall outcomes following STEMI have improved over time, it remains a notable health problem with significant morbidity and mortality (2). The in-hospital mortality rate associated with STEMI ranges from 8.4%–33.5%, and complications include heart failure, cardiogenic shock, malignant arrhythmias, ventricular free wall rupture and tamponade (2, 3). Early identification of patients who are at the highest risk of poorer outcomes after STEMI remains key in both mitigating these outcomes and the burden to the healthcare system.

The Thrombolysis In Myocardial Infarction (TIMI) and Global Registry of Acute Coronary Events (GRACE) scores are commonly used risk assessment tools for acute coronary syndrome, including STEMI (4, 5). However, treatment strategies for STEMI have evolved significantly since the creation of these scores and their applicability in this contemporary setting remains uncertain. The GRACE risk score encompasses both STEMI and non-ST-segment elevation myocardial infarction (NSTEMI) patients, thus limiting its suitability as a predictive model specifically for STEMI patients, who remain a higher risk cohort (5–7). Whilst the TIMI risk score targets STEMI patients, it has been developed from a cohort of patients managed predominantly with thrombolytic therapy, as opposed to the contemporary gold standard of primary percutaneous reperfusion (4, 8, 9). Moreover, these traditional risk scoring models may fail to capture nonlinear effects of significance and oversimplify intricate interrelations among variables (10).

Consequently, there remains a need for sophisticated predictive models that accurately predict outcomes in patients after STEMI, including mortality, intensive care unit (ICU) admission and left ventricular impairment, to allow for effective risk stratification, resource allocation and optimal patient management. Machine-learning (ML) is a branch of computer science dedicated to predicting outcomes in intricate datasets through various algorithms that iteratively learn from the data (11, 12). In contrast to conventional statistical methods, which dependent on predefined assumptions regarding data behaviour and preselected variables, ML algorithms empower the model to develop by recognizing and integrating underlying data patterns (13, 14). ML models have consistently exhibited significantly better performance when compared to traditional methods for predicting risks (10).

The aim of our study is to develop a highly interpretable and effective machine-learning based risk prediction algorithm for mortality, ICU admission and left ventricular ejection fraction less than 40% (the STEMI-ML Score).

## 2 Methods

### 2.1 Study cohort

We reviewed all consecutive patients presenting to our tertiary Australian centre for primary percutaneous coronary intervention (pPCI) or rescue PCI (following thrombolysis) for STEMI from July 2010 to December 2019. The study received approval from the Northern Sydney Local Health District Human Research Ethics Committee. All patients were treated with aspirin prior to PCI, unless intolerant of oral medications and all patients were given intra-arterial, therapeutic heparin at the start of the procedure. The use of glycoprotein IIb/IIIa inhibitors was at the discretion of the proceduralist.

### 2.2 Exposure and outcome variables

Patient electronic medical records were analysed to identify key demographic, clinical, biochemical, imaging, and procedural details. At the onset of coronary angiography, invasive hemodynamic parameters such as heart rate (HR) and aortic systolic blood pressure (SBP) were recorded and labelled as “starting HR” and “starting SBP”. Individual coronary angiograms were analysed to determine the presence and maturity of collaterals, which were then graded according to the Rentrop classification as grade 0 (absence of collateral filling), grade 1 [filling of side branches of the infarct-related artery (IRA)], grade 2 (partial filling of the epicardial vessel of the IRA) and grade 3 (complete filling of the IRA). Patients with Rentrop grade 0 or 1 collaterals were labelled as having poor collaterals, and patients with Rentrop grade 2 or 3 collaterals were labelled as having robust collaterals. Left ventricular function was evaluated using transthoracic echocardiogram after STEMI, or if unavailable, ventriculography at the time of the index procedure. The time at the onset of continuous chest pain to the time of acquisition of the first angiographic image during percutaneous coronary intervention was defined as the ischaemic time.

The primary outcomes were in-hospital mortality, intensive care unit (ICU) admission and left ventricular ejection fraction less than 40% (LVEF < 40%). In the included sites, STEMI patients are routinely admitted to the Coronary Care Unit (CCU) for ongoing care. There were no specific criteria for ICU admission. Generally, patients who require significant inotropic/vasopressor support (e.g., noradrenaline or adrenaline), intubation/mechanical ventilation, mechanical circulatory support (e.g., intra-aortic balloon pump or extracorporeal membrane oxygenation) or significant clinical instability prohibiting ongoing care in CCU are transferred to ICU.

### 2.3 Machine-learning process

**Figure 1:**
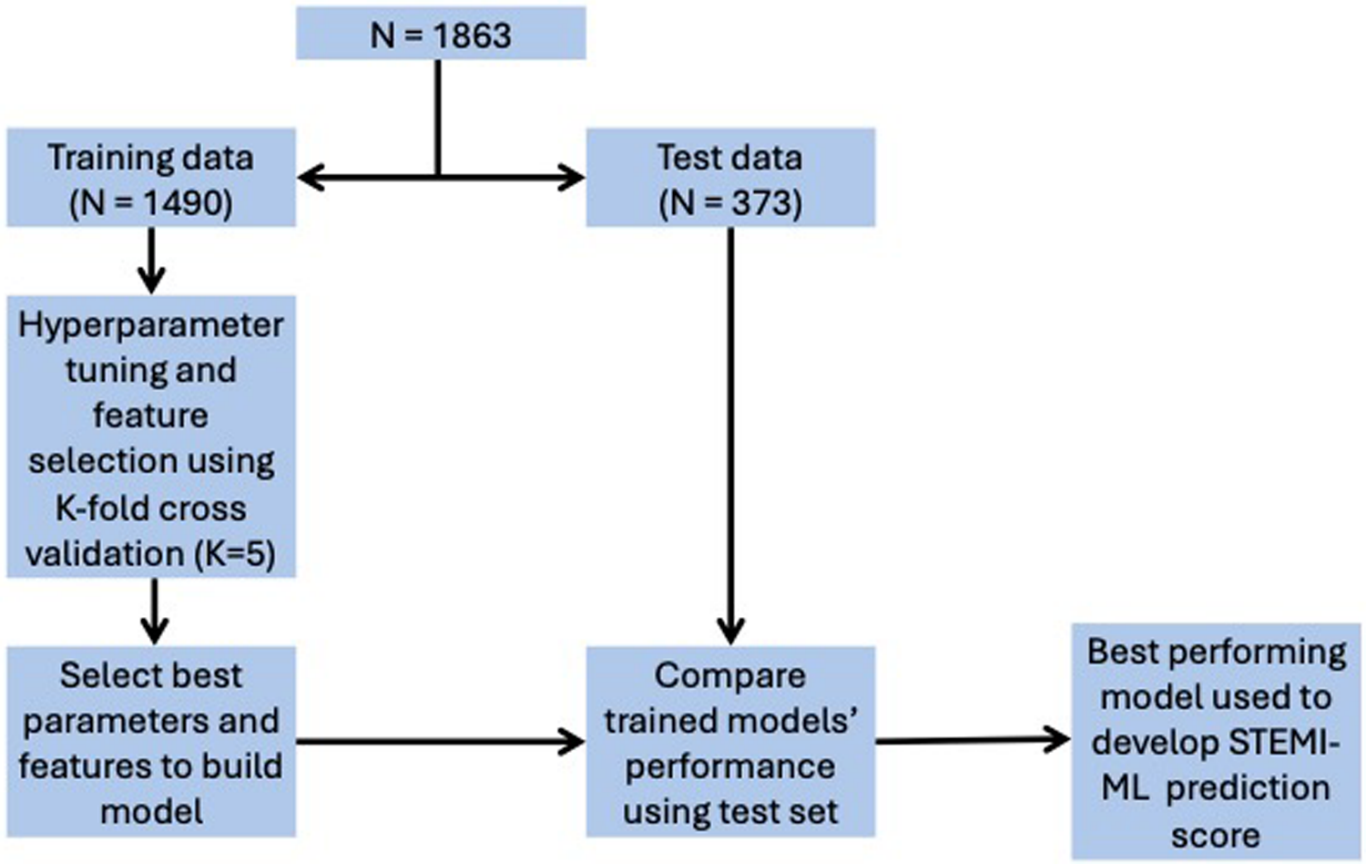
Overview of study methodology for data training and testing.

Data were separated into two subsets, with 80% used as training data for feature selection and training of model parameters and 20% used as test data to evaluate model performance. The methodology of this machine-learning process is shown in Figure 1. We used eight supervised-learning classification models to create risk prediction algorithms for the outcome variables of in-hospital mortality, ICU admission and LVEF < 40%. These models include three linear, logistic regression (LR) based models [least absolute shrinkage and selection operator (LASSO or L1), ridge (L2) and Elastic Net (EN)] and five non-linear, models [decision tree (DT), support vector machine (SVM), random forest (RF), AdaBoost (AB) and gradient boosting (GB)].

We oversampled the training dataset for each model by the minority class using SMOTENC, which is an extension of SMOTE (15). Missing values for each included feature were imputed by the median value and features were standardised by subtracting the mean and scaling to a variance of 1. Feature selection for LR was performed in a sequential forward fashion and a subset of features were selected in a greedy fashion, where the next best feature to add in the subset is based on the AUC cross validation score. The best 5/10/15/20/25/30(all) features as determined by the coefficient magnitude were used as features for other models. This particular process of extracting, ranking, and grouping features was utilised to promote model interpretability and usability.

Model hyperparameters were chosen using 5-fold cross validation with AUC as the scoring metric. After parameter tuning, each model was evaluated on the test set and the final model was chosen based on the AUC score. The final models were then calibrated by fitting a sigmoid regressor and isotonic regressor, and optimal calibration was evaluated using the the Brier score and calibration plot. The threshold for significance was established at 5% for all statistical tests. We performed our analyses with Python (version 3.7), and the methodology code can be found on: https://github.com/harisritharan/stemi_risk_prediction/blob/master/stemi%20model%20building.ipynb.

### 2.4 Application

To facilitate real-world use of our models, a pragmatic and user-friendly web application was built using Python Dash. Within this web application, the top-performing models for in-hospital mortality, ICU admission, and LVEF < 40% are incorporated, allowing users to manually select variables to generate a risk assessment score presented as a percentage.

## 3 Results

### 3.1 Study cohort

The total study cohort included 1,863 patients; 128 (6.9%) patients died in-hospital, 292 (19.0%) patients required ICU admission and 373 (20.8%) patients had LVEF < 40%. The mean age of patients was 64.9 ± 13.7 years and 77.1% were male. Additional study cohort characteristics are detailed in Table 1.

**Table 1:** Baseline characteristics.

	All patients	Proportion of missing values
Mean age (SD)—year	64.9 (13.7)	0%
Male–no. (%)	1,436 (77.1%)	0%
Body mass index (SD)—kg/m^2^	27.3 (4.8)	5.4%
Hypertension–no (%)	807 (46.4%)	6.7%
Hypercholesterolemia–no. (%)	677 (39.1%)	7.0%
Diabetes mellitus–no. (%)	278 (16.4%)	9.2%
Family history of coronary disease before age 50–no. (%)	458 (29.1%)	15.5%
Smoking history		19.2%
Never smoker–no. (%)	604 (40.2%)	
Ex-smoker–no. (%)	431 (28.7%)	
Current smoker–no. (%)	470 (31.3%)	
Pre-hospital cardiac arrest–no. (%)	218 (11.7%)	0%
Ischaemic time (SD)—min	484.5 (657.0)	11.1%
Starting heart rate (SD)–beats/min	79.8 (19.9)	0.6%
Starting systolic blood pressure (SD)—mmHg	122.7 (28.2)	0.9%
Previous stent–no. (%)	186 (10.1%)	7.5%
Culprit coronary artery		0%
Left anterior descending–no. (%)	885 (47.5%)	
Left circumflex–no. (%)	269 (14.4%)	
Right coronary–no. (%)	709 (38.1%)	
Robust collateral recruitment		0%
Yes (Rentrop grade 2 or 3)–no. (%)	399 (21.4%)	
No (Rentrop grade 0 or 1)–no. (%)	1,464 (78.6%)	
Thrombolysis in myocardial infarction flow pre-PCI		0%
0–no. (%)	1,096 (58.8%)	
1–no. (%)	156 (8.4%)	
2–no. (%)	511 (27.4%)	
3–no. (%)	100 (5.4%)	
Thrombolysis in myocardial infarction flow post-PCI		0%
0–no. (%)	21 (1.1%)	
1–no. (%)	29 (1.6%)	
2–no. (%)	138 (7.4%)	
3–no. (%)	1,675 (89.9%)	
Presence of chronic total occlusion in a remote vessel–no. (%)	116 (6.2%)	0%
Percutaneous coronary intervention performed–no. (%)	1,714 (92.0%)	0%
Number of stents (SD)–no.	1.2 (0.6)	0%
Length of stented segment (SD)—mm	30.1 (16.1)	8.0%
Glycoprotein IIb/IIIa inhibitor use–no. (%)	1,002 (54.8%)	1.8%
Inotrope use during procedure–no. (%)	269 (14.5%)	0.2%
Intra-aortic balloon pump (IABP) or extracorporeal membrane oxygenation (ECMO) use during procedure–no. (%)	44 (2.7%)	11.8%
Ventricular arrhythmia during procedure–no. (%)	124 (6.7%)	0.2%
In-hospital mortality–no. (%)	128 (6.9%)	0%
Intensive care unit admission–no. (%)	292 (15.7%)	1.4%
Left ventricular ejection fraction less than 40%–no. (%)	373 (20.8%)	3.8%

### 3.2 Predictive models for in-hospital mortality

**Figure 2:**
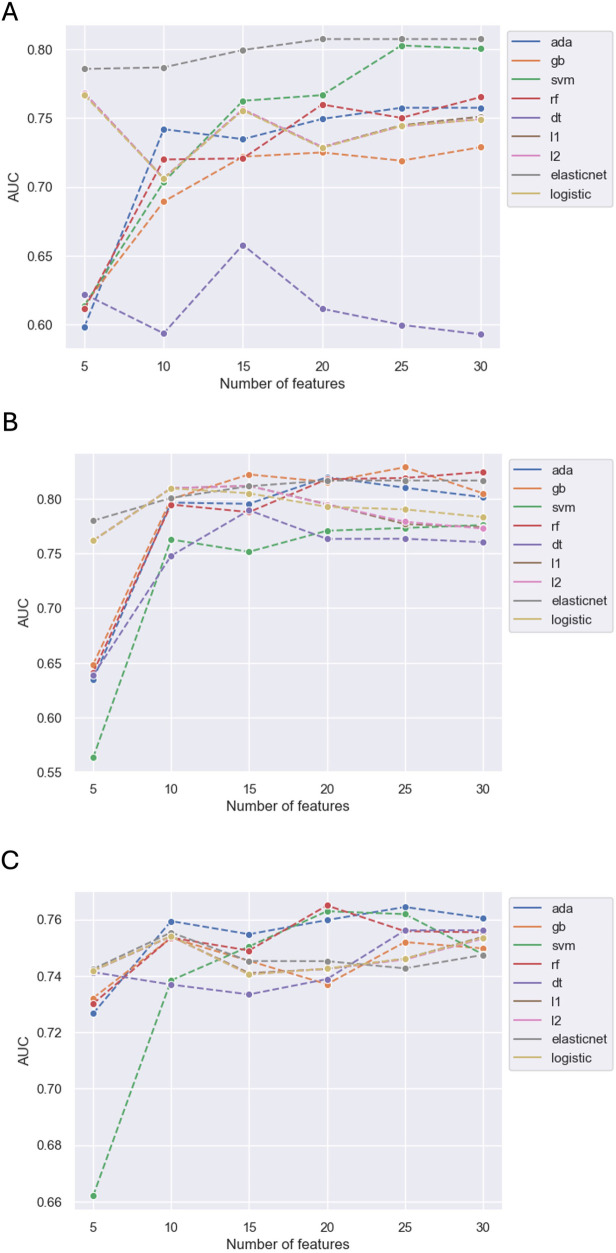
Predictive performance of models by number of variables for in-hospital mortality **(A)**, ICU admission **(B)** and LVEF < 40% **(C)**.

From the 8 ML algorithms applied, the model obtained by EN had the best performance for the outcome of in-hospital mortality when including 30 features (AUC 0.81), 25 features (AUC 0.81), 20 features (AUC 0.81), 15 features (AUC 0.80), 10 features (AUC 0.79) and 5 features (AUC 0.79). The changes in model performance across the 8 ML algorithms based on the number of included features is shown in Figure 2A.

When balancing model performance with ease of interpretability and useability, the model obtained by EN including 5 features was chosen as the final model (AUC 0.79, accuracy 0.74, precision 0.18, recall 0.68, f1 score 0.28). The performance of this EN model and its comparison against the performance of other models including 5 features derived by the remaining 7 ML algorithms is detailed in Supplementary Table S1; Supplementary Figure S1.

**Figure 3:**
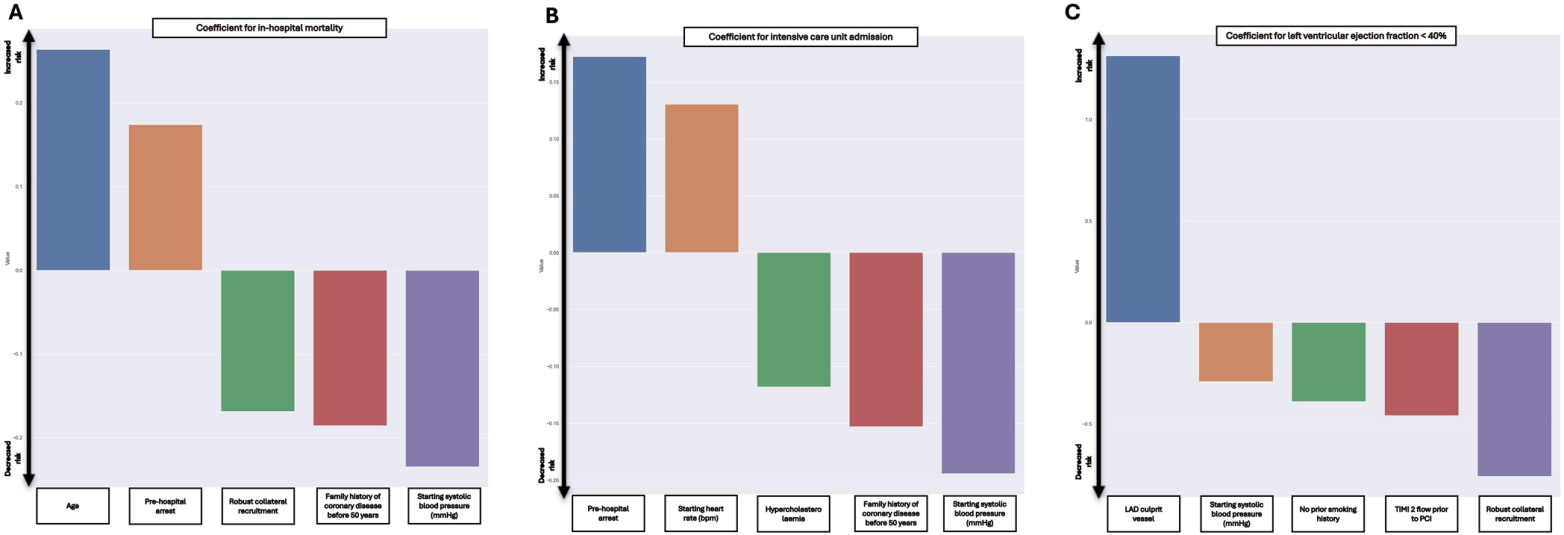
Included coefficients and weighting in final models for in-hospital mortality **(A)**, ICU admission **(B)** and LVEF < 40% **(C)**.

The results of the final model found that higher age and pre-hospital cardiac arrest were associated with increased in-hospital mortality. In contrast, the presence of robust collateral recruitment, a family history of coronary disease before age 50 and higher starting SBP were associated with lesser in-hospital mortality. The coefficients of the EN model were used to build the in-hospital mortality component of the STEMI-ML score (Figure 3A).

### 3.3 Predictive models for ICU admission

The model obtained by RF had the best performance for the outcome of ICU admission when including 30 features (AUC 0.82). The model obtained by GB performed best when including 25 features (AUC 0.83) and 15 features (AUC 0.82). The model obtained by AB had the best performance when including 20 features (AUC 0.82), and the model obtained by L2 had the best performance when including 10 variables (AUC 0.81). However, the model obtained by EN performed best when including 5 features (AUC 0.78). The changes in model performance across the 8 ML algorithms based on the number of included features is shown in Figure 2B.

When balancing model performance with ease of interpretability and useability, the model obtained by EN including 5 features was chosen as the final model (AUC 0.78, accuracy 0.76, precision 0.32, recall 0.67, f1 score 0.44). The performance of this EN model and its comparison against the performance of other models including 5 features derived by the remaining 7 ML algorithms is detailed in Supplementary Table S2; Supplementary Figure S2.

The results of the final model found that pre-hospital cardiac arrest and a higher starting HR were associated with increased ICU admission. However, pre-existing diagnosis of hypercholesterolemia, a family history of coronary disease before age 50 and higher starting SBP were associated with decreased ICU admission. The coefficients of this EN model were used to build the ICU admission component of the STEMI-ML score (Figure 3B).

### 3.4 Predictive models for LVEF < 40%

Out of the 8 ML algorithms, the model obtained by AB had the best performance for the outcome of LVEF < 40% when including 30 features (AUC 0.76), 25 features (AUC 0.76), 15 features (AUC 0.75) and 10 features (AUC 0.76). The model obtained by RF had the best performance when including 20 features (AUC 0.76). However, when including 5 features (AUC 0.74), the model obtained by EN had the best performance and this was marginally lower than the AB and RF models that included more features. The changes in model performance across the 8 ML algorithms based on the number of features included is shown in Figure 2C.

When balancing model performance with ease of interpretability and useability, the model obtained by EN including 5 features was chosen as the final model (AUC 0.74, accuracy 0.72, precision 0.44, recall 0.64, f1 score 0.52). The performance of this EN model and its comparison against the performance of other models including 5 features derived by the remaining 7 ML algorithms is detailed in Supplementary Table S3; Supplementary Figure S3.

The results of the final model found that the left anterior descending coronary artery as the culprit vessel was associated increased likelihood of LVEF < 40%. However, higher starting SBP, current smoking status, TIMI flow 2 pre-PCI and the presence of robust collateral recruitment were associated with decreased likelihood of LVEF < 40%. The coefficients of this EN model were used to build the LVEF < 40% component of the STEMI-ML score (Figure 3C).

### 3.5 The STEMI-ML score application

**Figure 4:**
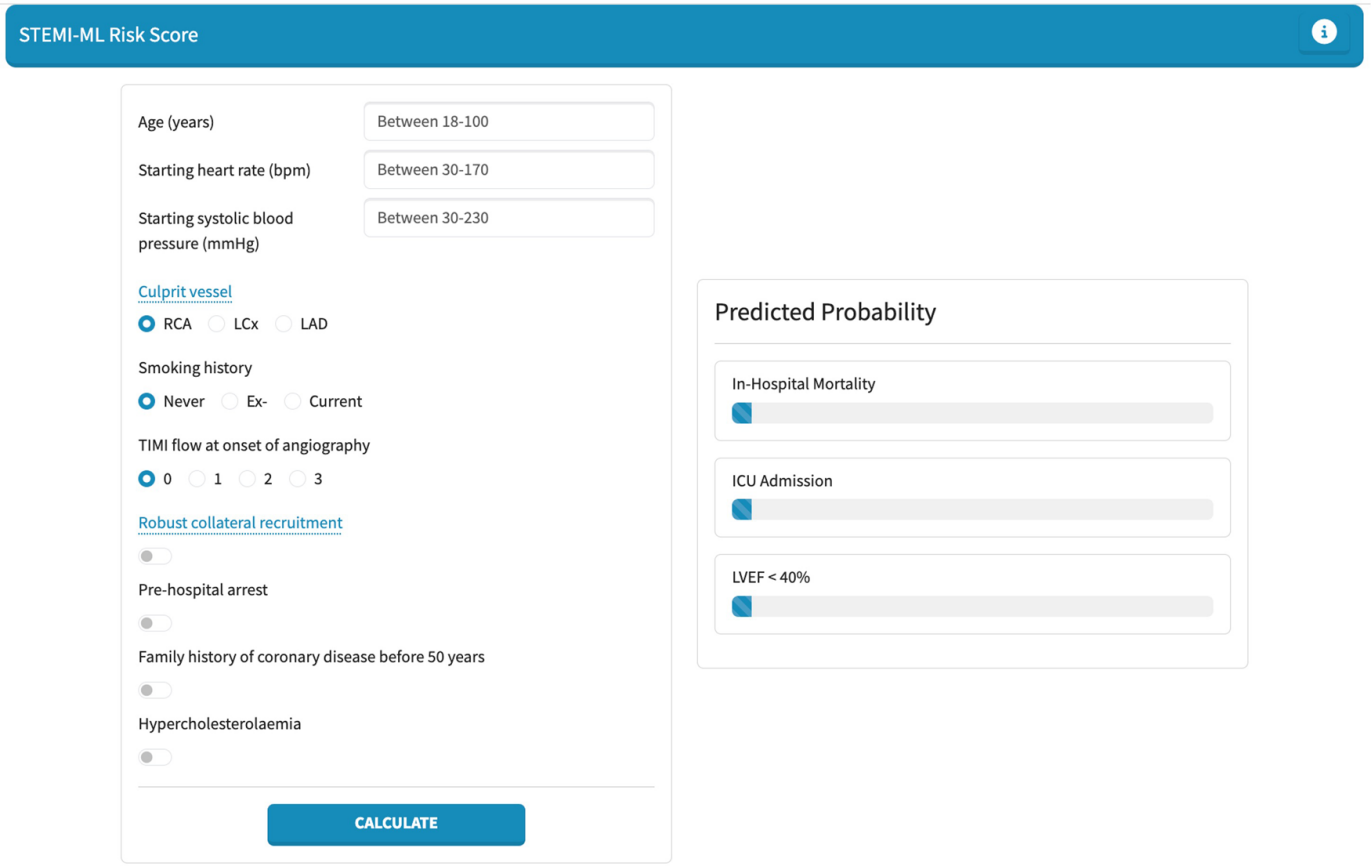
STEMI-ML risk score web application.

Finally, we developed a web-based application for the individual probability for in-hospital mortality, ICU admission and LVEF < 40% in STEMI patients. This web application is shown in Figure 4 and is available at: https://stemi-ml-score.onrender.com.

## 4 Discussion

We present dedicated machine-learning based risk prediction models for in-hospital mortality, ICU admission and LVEF < 40% in STEMI patients. These models are practical in their design and use data that is routinely collected during and in the lead up to PCI; thereby allowing for risk prediction immediately post-PCI. A criticism of existing prediction models is the large number of variables included and the limited useability in this context. We have pragmatically included five variables in all our models and have still achieved highly effective predictive capabilities with AUC greater than 0.7 and comparable if not superior efficacy to existing risk scores (16, 17). Moreover, we have developed a user-friendly, web-based application, the “STEMI-ML Score”, to assist clinicians at the bedside in risk stratifying patients in real-time: https://stemi-ml-score.onrender.com.

The presence of robust coronary collaterals has previously been reported as a protective factor for in-hospital mortality and left ventricular function in STEMI patients, however existing risk models do not consider it as one of their exposure variables (18–20). This significant impact of robust coronary collaterals on in-hospital outcomes is again reflected in our study, where it features in our final models for mortality and left ventricular impairment. The inclusion of variables such as coronary collateral circulation presents a major strength of our study and methodology with a machine-learning approach, wherein a larger number of variables can be considered, and key novel signals can be appreciated.

Whilst patients with a family history of coronary disease are at increased risk of developing a myocardial infarction, observational studies have conversely demonstrated lower in-hospital mortality and adverse clinical events in this group. A retrospective, observational study of 2,123,492 STEMI admissions demonstrated significantly lower odds of in-hospital mortality in patients with a family history of coronary artery disease compared to patients without [1.4% vs. 8.1%; aOR 0.42, 95% confidence interval (CI): 0.41–0.44; *P* < 0.001] (21). The purported mechanism for this difference was the influence of family history on heightened patient awareness of cardiovascular health and increased patient focus on pharmacological and non-pharmacological modalities of cardiovascular risk reduction (21). Our final models for in-hospital mortality and ICU admission find family history of coronary disease before age 50 to be a significant protective factor.

Several studies have investigated the cholesterol paradox in patients with acute coronary syndromes and NSTEMI or STEMI alone (22, 23). While hypercholesterolemia is a well-known risk factor for STEMI, studies have demonstrated lower risk of adverse events following STEMI in patients with hypercholesterolemia (22, 24). A retrospective, observational study including 11,543 STEMI patients and 8,470 NSTEMI patients demonstrated lower adverse events in patients with higher low-density lipoprotein-C and triglyceride levels (24). Similarly, the smoker's paradox has also been investigated in several studies, with a lower risk of adverse events noted especially in current smokers following STEMI (25, 26). This smoker's paradox may be explained by the younger age and fewer cardiovascular risk factors in smokers compared with non-smokers (27). Both the cholesterol paradox and smoker's paradox are demonstrated in our final models, with hypercholesterolemia a protective factor for ICU admission and current smoking a protective factor for left ventricular impairment.

Higher age and pre-hospital cardiac arrest are well-established predictors of poor outcomes in STEMI patients and are seen in our models for in-hospital mortality and ICU admission (28–30). A higher starting SBP is also an established protective predictor of outcomes in STEMI patients and features in all our final models (31). However, a higher starting heart rate has been associated with poorer outcomes in STEMI patients and was similarly a predictor for ICU admission in our study (32). Left ventricular impairment with an ejection fraction less than 40% was seen in 20.8% of patients in our study, and a culprit lesion in the left anterior descending artery (LAD) was a significant predictor of this outcome, likely mediated by the large myocardial territory supplied by the LAD.

Our study has some important limitations. Whilst our model was validated on an internal test dataset, external validation on another dataset would be ideal and necessary prior to consideration of widespread use. External validation also would be ideal from the perspective of validating the generalisability of our study, which involved a single centre only. Furthermore, our dataset exhibited an imbalance in the outcome variables, which is a common challenge encountered in the construction of machine-learning-based risk prediction algorithms for medical applications where the outcomes may be less common. To address this issue, we employed oversampling of the minority class during the training of each model, and thereby mitigated the impact of this imbalance on the algorithm's performance. Finally, the exposure variables included in the analysis were not comprehensive, and missing variables, although imputed and uncommon, may influence the outcomes.

## 5 Conclusion

We present a highly interpretable and effective machine-learning based risk prediction algorithm to predict in-hospital mortality, ICU admission and LVEF < 40% in STEMI patients: the STEMI-ML Score. This may assist in the risk stratification, individualised monitoring, and management of STEMI patients.

## Data Availability

The original contributions presented in the study are included in the article, further inquiries can be directed to the corresponding author.
