# Supplementary material for: Machine-learning based risk prediction of in-hospital outcomes following STEMI: the STEMI-ML score
Source: Front Cardiovasc Med. 2024 Oct 10;11:1454321. doi: 10.3389/fcvm.2024.1454321 (PMC11499125; doi:10.3389/fcvm.2024.1454321)

Supplementary Material

1 Supplementary Data, Figures and Tables

Table S1: Model performance in the prediction of in-hospital mortality

Model	Number of features	AUC	Accuracy	Precision	Recall	F1 score
L1	5	0.7682194616977230	0.7345844504021450	0.1619047619047620	0.6071428571428570	0.25563909774436100
	10	0.7062111801242240	0.7560321715817690	0.15384615384615400	0.5	0.23529411764705900
	15	0.7562111801242240	0.7855227882037530	0.175	0.5	0.25925925925925900
	20	0.7289855072463770	0.8016085790884720	0.16176470588235300	0.39285714285714300	0.22916666666666700
	25	0.7448240165631470	0.8123324396782840	0.19117647058823500	0.4642857142857140	0.27083333333333300
	30	0.7511387163561080	0.8150134048257370	0.19402985074626900	0.4642857142857140	0.2736842105263160
L2	5	0.7684265010351970	0.7345844504021450	0.1619047619047620	0.6071428571428570	0.25563909774436100

	10	0.7061076604554870	0.7560321715817690	0.15384615384615400	0.5	0.23529411764705900
	15	0.7565217391304350	0.7855227882037530	0.175	0.5	0.25925925925925900
	20	0.7290890269151140	0.806970509383378	0.16666666666666700	0.39285714285714300	0.2340425531914890
	25	0.7439958592132510	0.8123324396782840	0.19117647058823500	0.4642857142857140	0.27083333333333300
	30	0.7493788819875780	0.8123324396782840	0.2	0.5	0.28571428571428600
**EN**	**5**	**<u>0.7858178053830230</u>**	**0.7426273458445040**	**0.1792452830188680**	**0.6785714285714290**	**0.2835820895522390**
	10	0.7869565217391300	0.7828418230563000	0.2087912087912090	0.6785714285714290	0.31932773109243700
	15	0.7995859213250520	0.7801608579088470	0.20652173913043500	0.6785714285714290	0.31666666666666700
	20	0.8075569358178050	0.7908847184986600	0.2222222222222220	0.7142857142857140	0.3389830508474580
	25	0.8075569358178050	0.7908847184986600	0.2222222222222220	0.7142857142857140	0.3389830508474580

	30	0.8075569358178050	0.7908847184986600	0.2222222222222220	0.7142857142857140	0.3389830508474580
SVM	5	0.6135610766045550	0.6300268096514750	0.09558823529411760	0.4642857142857140	0.15853658536585400
	10	0.703416149068323	0.7319034852546920	0.10869565217391300	0.35714285714285700	0.16666666666666700
	15	0.7626293995859210	0.8176943699731900	0.1875	0.42857142857142900	0.26086956521739100
	20	0.7667701863354040	0.8257372654155500	0.1864406779661020	0.39285714285714300	0.25287356321839100
	25	0.8028985507246380	0.8632707774798930	0.23255813953488400	0.35714285714285700	0.28169014084507000
	30	0.8005175983436850	0.8659517426273460	0.225	0.32142857142857100	0.2647058823529410
DT	5	0.6219461697722570	0.6300268096514750	0.09558823529411760	0.4642857142857140	0.15853658536585400
	10	0.5933747412008280	0.6032171581769440	0.0945945945945946	0.5	0.1590909090909090
	15	0.6578157349896480	0.6568364611260050	0.109375	0.5	0.1794871794871800

	20	0.6112836438923400	0.6514745308310990	0.08870967741935480	0.39285714285714300	0.14473684210526300
	25	0.5995859213250520	0.6541554959785520	0.08264462809917360	0.35714285714285700	0.1342281879194630
	30	0.5925983436853000	0.6085790884718500	0.09027777777777780	0.4642857142857140	0.15116279069767400
RF	5	0.6111801242236030	0.6300268096514750	0.09558823529411760	0.4642857142857140	0.15853658536585400
	10	0.719927536231884	0.7613941018766760	0.13253012048192800	0.39285714285714300	0.1981981981981980
	15	0.720703933747412	0.7050938337801610	0.15254237288135600	0.6428571428571430	0.2465753424657530
	20	0.7599378881987580	0.8284182305630030	0.2	0.42857142857142900	0.27272727272727300
	25	0.7502070393374740	0.8257372654155500	0.19672131147541000	0.42857142857142900	0.2696629213483150
	30	0.765320910973085	0.8498659517426270	0.22	0.39285714285714300	0.28205128205128200
AB	5	0.5980848861283640	0.6085790884718500	0.09027777777777780	0.4642857142857140	0.15116279069767400

	10	0.7419772256728780	0.8176943699731900	0.21428571428571400	0.5357142857142860	0.3061224489795920
	15	0.7347308488612840	0.7882037533512060	0.17721518987341800	0.5	0.2616822429906540
	20	0.7494306418219460	0.806970509383378	0.19444444444444400	0.5	0.28
	25	0.7575569358178050	0.7908847184986600	0.1951219512195120	0.5714285714285710	0.29090909090909100
	30	0.7575569358178050	0.7908847184986600	0.1951219512195120	0.5714285714285710	0.29090909090909100
GB	5	0.6141821946169770	0.6300268096514750	0.09558823529411760	0.4642857142857140	0.15853658536585400
	10	0.6892857142857140	0.6943699731903490	0.12931034482758600	0.5357142857142860	0.20833333333333300
	15	0.7219461697722570	0.7265415549597860	0.1509433962264150	0.5714285714285710	0.23880597014925400
	20	0.7250517598343690	0.7292225201072390	0.1588785046728970	0.6071428571428570	0.2518518518518520
	25	0.719047619047619	0.7426273458445040	0.15306122448979600	0.5357142857142860	0.23809523809523800

	30	0.72888198757 76400	0.75603217158 17690	0.168421052631 57900	0.571428571428 5710	0.260162601626 0160

Table S2: Model performance in the prediction of intensive care unit (ICU) admission

Model	Number of features	AUC	Accuracy	Precision	Recall	F1 score
L1	5	0.7618670886075950	0.7282608695652170	0.29310344827586200	0.6538461538461540	0.40476190476190500
	10	0.8097005842259010	0.7418478260869570	0.3162393162393160	0.7115384615384620	0.4378698224852070
	15	0.8118914313534570	0.7880434782608700	0.3673469387755100	0.6923076923076920	0.48000000000000000
	20	0.7951557935735150	0.7853260869565220	0.35789473684210500	0.6538461538461540	0.4625850340136050
	25	0.7768987341772150	0.7853260869565220	0.3548387096774190	0.6346153846153850	0.4551724137931030
	30	0.7740993184031160	0.7880434782608700	0.358695652173913	0.6346153846153850	0.45833333333333300
L2	5	0.7616845180136320	0.7282608695652170	0.29310344827586200	0.6538461538461540	0.40476190476190500
	10	0.809761441090555	0.7418478260869570	0.3162393162393160	0.7115384615384620	0.4378698224852070
	15	0.8119522882181110	0.7880434782608700	0.3673469387755100	0.6923076923076920	0.48000000000000000

	20	0.7947297955209350	0.782608695652174	0.35106382978723400	0.6346153846153850	0.4520547945205480
	25	0.7790895813047710	0.7989130434782610	0.375	0.6346153846153850	0.4714285714285710
	30	0.7727604673807210	0.7880434782608700	0.358695652173913	0.6346153846153850	0.45833333333333300
**EN**	**5**	**0.7800024342745860**	**0.7554347826086960**	**0.32407407407407400**	**0.6730769230769230**	**0.4375**
	10	0.800815481986368	0.7744565217391310	0.3523809523809520	0.7115384615384620	0.4713375796178350
	15	0.8115262901655310	0.7880434782608700	0.375	0.75	0.5
	20	0.8167599805258030	0.7880434782608700	0.375	0.75	0.5
	25	0.8167599805258030	0.7880434782608700	0.375	0.75	0.5
	30	0.8167599805258030	0.7880434782608700	0.375	0.75	0.5
SVM	5	0.5632302823758520	0.5163043478260870	0.18181818181818200	0.6923076923076920	0.28800000000000000

	10	0.7627799415774100	0.7255434782608700	0.28695652173913000	0.6346153846153850	0.3952095808383230
	15	0.7515518500486860	0.7581521739130440	0.29213483146067400	0.5	0.36879432624113500
	20	0.7708130477117820	0.7717391304347830	0.30952380952381000	0.5	0.3823529411764710
	25	0.7733690360272640	0.7961956521739130	0.3466666666666670	0.5	0.4094488188976380
	30	0.7760163096397270	0.8016304347826090	0.36	0.5192307692307690	0.4251968503937010
DT	5	0.6384493670886080	0.46467391304347800	0.17488789237668200	0.75	0.2836363636363640
	10	0.7479004381694250	0.8342391304347830	0.44155844155844200	0.6538461538461540	0.5271317829457360
	15	0.7895569620253160	0.8396739130434780	0.45454545454545500	0.6730769230769230	0.5426356589147290
	20	0.763327653359299	0.8396739130434780	0.45454545454545500	0.6730769230769230	0.5426356589147290
	25	0.7634797955209350	0.8396739130434780	0.45454545454545500	0.6730769230769230	0.5426356589147290

	30	0.7603456669912370	0.8369565217391310	0.44871794871794900	0.6730769230769230	0.5384615384615380
RF	5	0.6410966407010710	0.4945652173913040	0.18981481481481500	0.7884615384615380	0.3059701492537310
	10	0.794760223953262	0.7445652173913040	0.33064516129032300	0.7884615384615380	0.46590909090909100
	15	0.7878529698149950	0.779891304347826	0.3592233009708740	0.7115384615384620	0.47741935483871000
	20	0.8179771178188900	0.7853260869565220	0.3516483516483520	0.6153846153846150	0.4475524475524480
	25	0.819072541382668	0.8070652173913040	0.39080459770114900	0.6538461538461540	0.4892086330935250
	30	0.8246105160662120	0.7934782608695650	0.375	0.6923076923076920	0.48648648648648600
AB	5	0.6349196689386560	0.453804347826087	0.1829787234042550	0.8269230769230770	0.29965156794425100
	10	0.796585929892892	0.7771739130434780	0.3611111111111110	0.75	0.48750000000000000
	15	0.7954296494644600	0.7472826086956520	0.3247863247863250	0.7307692307692310	0.4497041420118340

	20	0.8197723953261930	0.7690217391304350	0.35135135135135100	0.75	0.47852760736196300
	25	0.8101874391431350	0.7690217391304350	0.35135135135135100	0.75	0.47852760736196300
	30	0.8015457643622200	0.7445652173913040	0.3157894736842110	0.6923076923076920	0.43373493975903600
GB	5	0.6484907497565730	0.47554347826087000	0.18385650224215200	0.7884615384615380	0.2981818181818180
	10	0.7997504868549170	0.7907608695652170	0.3786407766990290	0.75	0.5032258064516130
	15	0.8223283836416750	0.8233695652173910	0.42857142857142900	0.75	0.5454545454545450
	20	0.815421129503408	0.782608695652174	0.36792452830188700	0.75	0.4936708860759490
	25	0.8289313534566700	0.7989130434782610	0.3877551020408160	0.7307692307692310	0.5066666666666670
	30	0.8050754625121710	0.8043478260869570	0.391304347826087	0.6923076923076920	0.5

Table S3: Model performance in the prediction of left ventricular ejection fraction less than 40% (LVEF < 40%)

Model	Number of features	AUC	Accuracy	Precision	Recall	F1 score
L1	5	0.7417965367965370	0.724233983286908	0.43902439024390200	0.6428571428571430	0.5217391304347830
	10	0.753982683982684	0.7604456824512530	0.49038461538461500	0.6071428571428570	0.5425531914893620
	15	0.740952380952381	0.7325905292479110	0.4375	0.5	0.4666666666666670
	20	0.7424242424242430	0.7298050139275770	0.43564356435643600	0.5238095238095240	0.47567567567567600
	25	0.7460173160173160	0.7270194986072420	0.43137254901960800	0.5238095238095240	0.4731182795698930
	30	0.7538095238095240	0.7353760445682450	0.44761904761904800	0.5595238095238100	0.49735449735449700
L2	5	0.7418831168831170	0.724233983286908	0.43902439024390200	0.6428571428571430	0.5217391304347830
	10	0.7540259740259740	0.7576601671309190	0.4857142857142860	0.6071428571428570	0.5396825396825400
	15	0.7405194805194810	0.7325905292479110	0.4375	0.5	0.4666666666666670

	20	0.7425108225108230	0.7298050139275770	0.43564356435643600	0.5238095238095240	0.47567567567567600
	25	0.7457575757575760	0.7270194986072420	0.43137254901960800	0.5238095238095240	0.4731182795698930
	30	0.7531601731601730	0.7325905292479110	0.44339622641509400	0.5595238095238100	0.49473684210526300
**EN**	**5**	**0.7424025974025970**	**0.7214484679665740**	**0.43548387096774200**	**0.6428571428571430**	**0.5192307692307690**
	10	0.7553679653679650	0.7576601671309190	0.48598130841121500	0.6190476190476190	0.5445026178010470
	15	0.7452380952380950	0.7409470752089140	0.4563106796116510	0.5595238095238100	0.5026737967914440
	20	0.7451948051948050	0.7409470752089140	0.4563106796116510	0.5595238095238100	0.5026737967914440
	25	0.7426406926406930	0.7409470752089140	0.4536082474226800	0.5238095238095240	0.4861878453038670
	30	0.7474025974025970	0.754874651810585	0.47959183673469400	0.5595238095238100	0.5164835164835170
SVM	5	0.661948051948052	0.7520891364902510	0.4666666666666670	0.4166666666666670	0.44025157232704400

	10	0.7383549783549780	0.7325905292479110	0.4318181818181820	0.4523809523809520	0.4418604651162790
	15	0.7503896103896100	0.7325905292479110	0.4333333333333300	0.4642857142857140	0.4482758620689660
	20	0.762900432900433	0.7437325905292480	0.4545454545454550	0.476190476190476	0.4651162790697670
	25	0.7618614718614720	0.7520891364902510	0.4705882352941180	0.476190476190476	0.4733727810650890
	30	0.7476190476190480	0.7493036211699160	0.4625	0.440476190476190	0.4512195121951220
DT	5	0.7412987012987010	0.754874651810585	0.4772727272727270	0.5	0.4883720930232560
	10	0.736883116883117	0.7409470752089140	0.4594594594594600	0.6071428571428570	0.5230769230769230
	15	0.7333982683982690	0.7493036211699160	0.46875	0.5357142857142860	0.5
	20	0.7388311688311690	0.7520891364902510	0.4736842105263160	0.5357142857142860	0.5027932960893850
	25	0.7561255411255410	0.7409470752089140	0.4594594594594600	0.6071428571428570	0.5230769230769230

	30	0.7561255411255410	0.7409470752089140	0.4594594594594600	0.6071428571428570	0.5230769230769230
RF	5	0.7301298701298700	0.7075208913649030	0.4244604316546760	0.7023809523809520	0.5291479820627800
	10	0.7535281385281390	0.7270194986072420	0.4406779661016950	0.6190476190476190	0.5148514851485150
	15	0.7489610389610390	0.7075208913649030	0.4117647058823530	0.5833333333333330	0.4827586206896550
	20	0.7649350649350650	0.7353760445682450	0.45132743362831900	0.6071428571428570	0.5177664974619290
	25	0.7558008658008660	0.7075208913649030	0.416	0.6190476190476190	0.4976076555023920
	30	0.7554112554112550	0.7381615598885790	0.45614035087719300	0.6190476190476190	0.5252525252525250
AB	5	0.7266233766233770	0.7047353760445680	0.4126984126984130	0.6190476190476190	0.49523809523809500
	10	0.7593939393939390	0.7520891364902510	0.47706422018348600	0.6190476190476190	0.538860103626943
	15	0.7547402597402600	0.7409470752089140	0.4594594594594600	0.6071428571428570	0.5230769230769230

	20	0.7597619047619050	0.7409470752089140	0.46017699115044200	0.6190476190476190	0.5279187817258880
	25	0.7643939393939390	0.754874651810585	0.48148148148148100	0.6190476190476190	0.5416666666666670
	30	0.7605194805194800	0.7437325905292480	0.46296296296296300	0.5952380952380950	0.5208333333333330
GB	5	0.7321212121212120	0.6852367688022280	0.4013605442176870	0.7023809523809520	0.5108225108225110
	10	0.7534632034632040	0.7075208913649030	0.4198473282442750	0.6547619047619050	0.5116279069767440
	15	0.7453246753246750	0.724233983286908	0.4380165289256200	0.6309523809523810	0.5170731707317070
	20	0.7370129870129870	0.7186629526462400	0.42201834862385300	0.5476190476190480	0.4766839378238340
	25	0.751948051948052	0.6963788300835660	0.4074074074074070	0.6547619047619050	0.502283105022831
	30	0.7497186147186150	0.7186629526462400	0.4330708661417320	0.6547619047619050	0.5213270142180100

Figure S1: Receiver operating characteristic curves for all models including 5 features for in-hospital mortality (left) and chosen EN model (right)

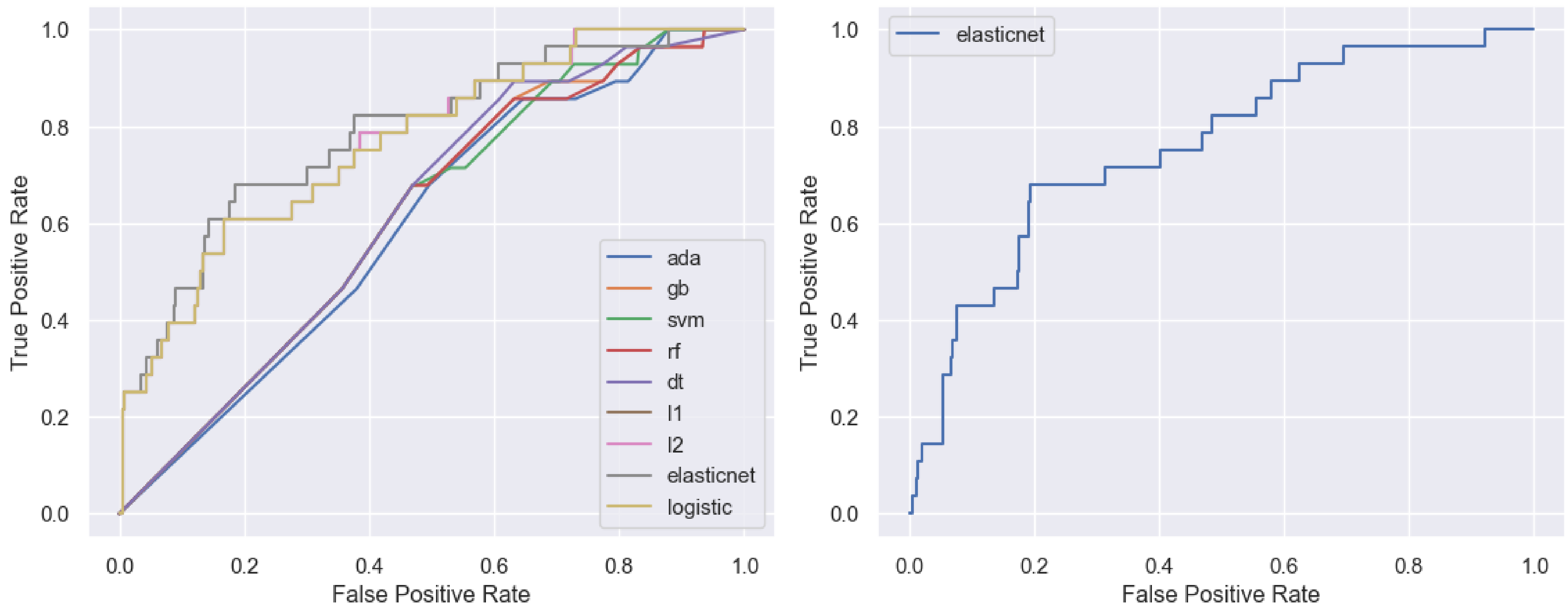

Figure S2: Receiver operating characteristic curves for all models including 5 features for ICU admission (left) and chosen EN model (right)

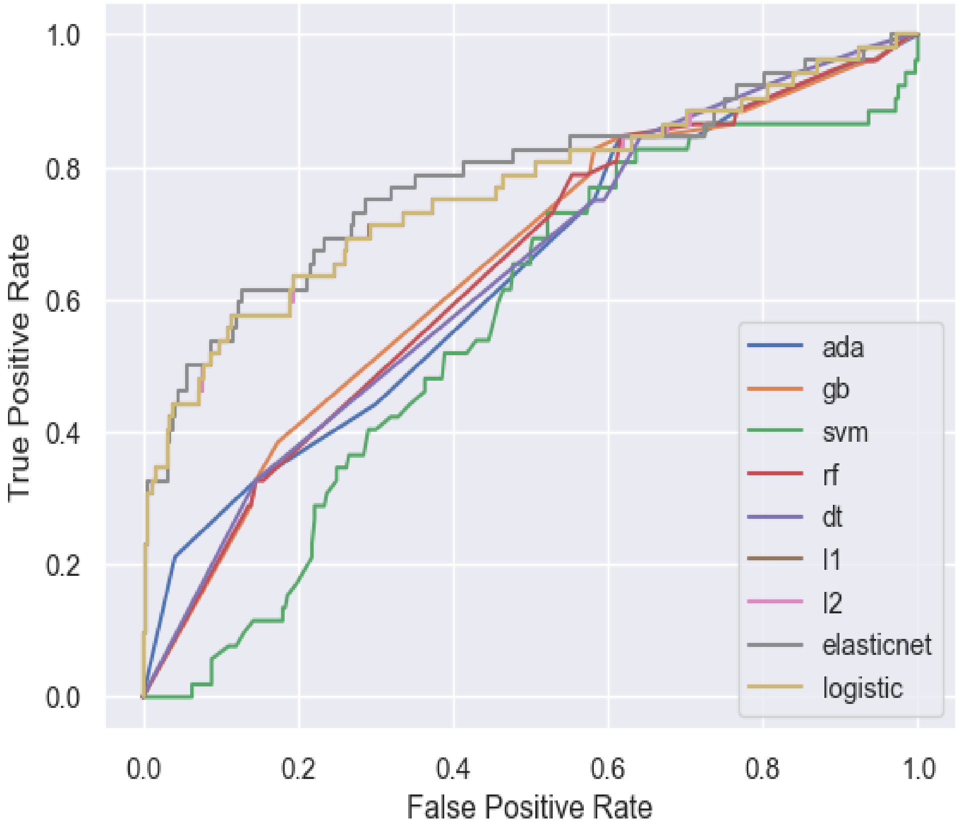

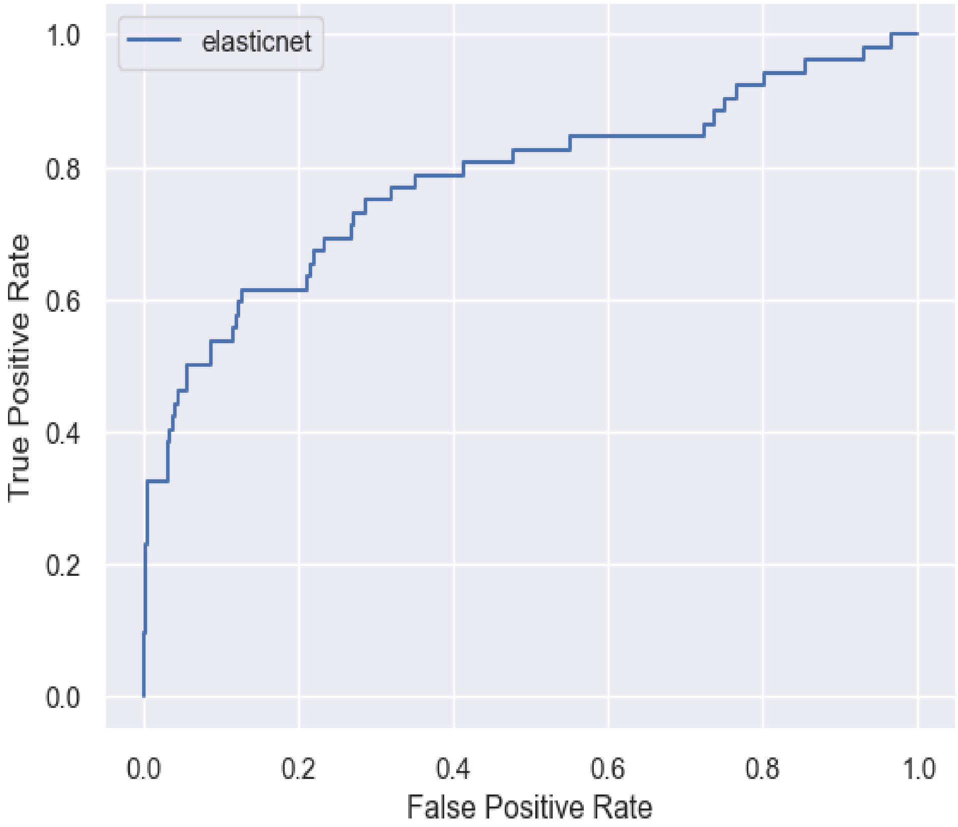

Figure S3: Receiver operating characteristic curves for all models including 5 features for LVEF < 40% (left) and chosen EN model (right)

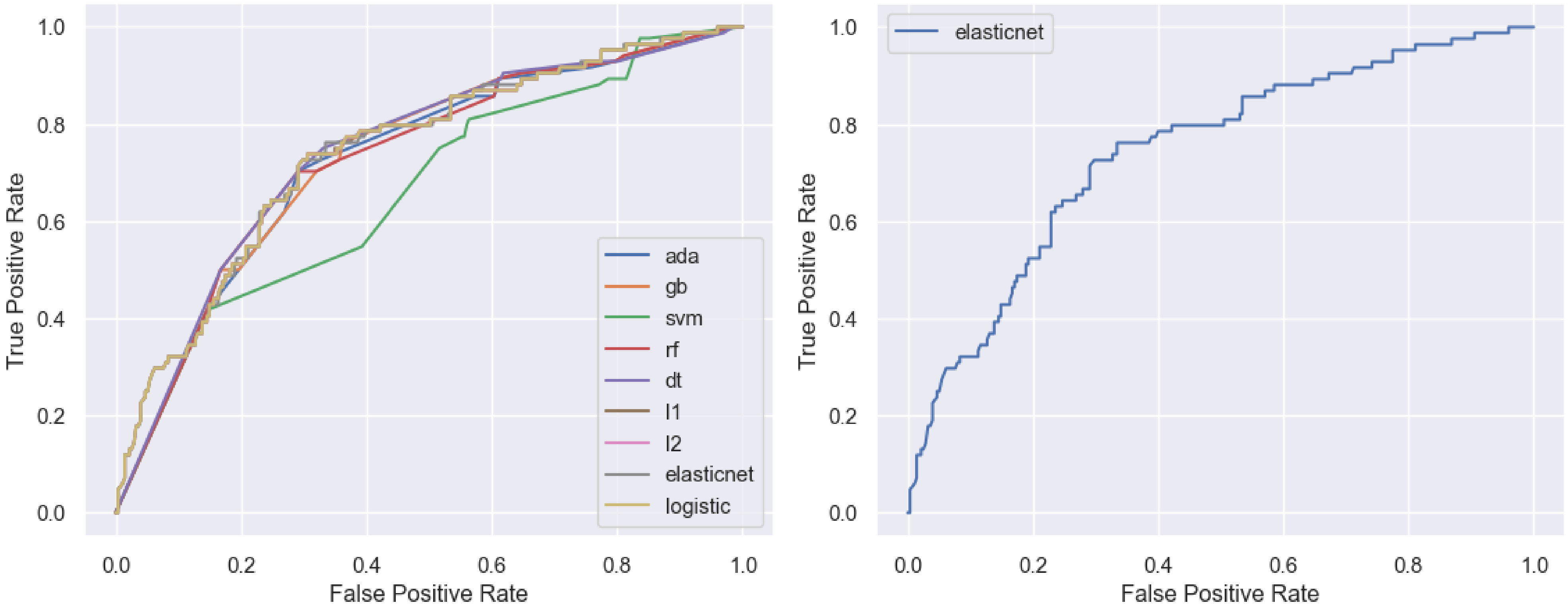

Supplement: Supplementary file 1 [file Datasheet1.pdf]
